# Perspectives of obese children and their parents on lifestyle behavior change: a qualitative study

**DOI:** 10.1186/s12966-015-0263-8

**Published:** 2015-08-19

**Authors:** AAH Schalkwijk, SDM Bot, L. de Vries, MJ Westerman, G. Nijpels, PJM Elders

**Affiliations:** Department of General Practice & Elderly Care Medicine and the EMGO Institute for Health and Care Research, VU University Medical Center, Van der Boechorststraat 7, 1081 BT Amsterdam, The Netherlands; Department of Methodology and Statistics, Institute of Health Sciences and the EMGO Institute for Health and Care Research, VU University, Amsterdam, The Netherlands

**Keywords:** Childhood obesity, Lifestyle intervention, Parenting, Social context, Qualitative research, General practitioner

## Abstract

**Background:**

In order to improve and optimize future behavioral family lifestyle intervention programs, more information on the perceptions of obese children and their parents of these programs is needed. As such, the aim of this qualitative study is 1) to explore the expectations of obese children and their parents in relation to lifestyle interventions; 2) to identify barriers to making lifestyle changes that parents and children face within their social context (within the family, at school and amongst friends and peers) as well as the things that facilitate these changes and 3) to identify the needs of obese children and their parents in the context of a lifestyle intervention.

**Methods:**

A qualitative study using semi-structured interviews was conducted. Interviewees were participants in a lifestyle intervention program in the Netherlands.

**Results:**

Eighteen children (mean age 10 years) and 24 parents were interviewed. The respondents expected to lose weight by being physically active or by eating healthily. Parents struggled with adopting and adhering to new rules and the absence of support of family members. Children struggled with inconsistent parenting and a lack of support from their parents. Bullying experienced at school impeded the children in their ability to make the necessary changes. Support from peers, on the other hand, stimulated their progress. Parents identified the need for the general practitioner to discuss overweight in a non-offensive way and to show an interest in the process of weight loss.

**Conclusions:**

Participants in a lifestyle behavior intervention program benefit from parental support and help from their (extended) family, peers and friends. They would also profit from the sustained involvement of their general practitioner in assisting in the maintenance of lifestyle behavior changes.

**Electronic supplementary material:**

The online version of this article (doi:10.1186/s12966-015-0263-8) contains supplementary material, which is available to authorized users.

## Background

Over the past few decades, childhood overweight and obesity has become increasingly prevalent around the world [1]. In the Netherlands, overweight and obesity in children have more than doubled since 1980. In 2009, the prevalence of overweight and obese children was 12.8 and 1.8 % respectively for boys and 14.8 and 2.2 % respectively for girls (2–21 years) [2].

As childhood obesity is associated with serious co-morbidities [3–6] and psychosocial problems [4, 7], early treatment and prevention are important to ensuring sustained health at a later age.

The internationally recommended treatment of childhood obesity includes a behavioral family lifestyle intervention program with dietary and physical activity advice and a family-targeted focus in children under 12 years of age [8, 9]. However, studies included in the Cochrane review on interventions for treating obesity in children suggest that their success is limited in terms of addressing childhood obesity over the long term [9]. Furthermore, the authors concluded that most studies were underpowered and were subject to high drop-out rates. While the two reviews referenced indicate the importance of behavioral family lifestyle intervention, neither explicitly deals with the influence of the participant’s social network on the intervention. Previous research by Pocok et al. found that parents’ perceptions of healthy behaviors to prevent childhood overweight are complex and are influenced at many levels [10]. Furthermore, intergenerational influences on parental health beliefs and knowledge suggest that health promotion strategies may be more effective if they are directed at the wider family, rather than at parents alone [10]. In addition, Christakis et al. suggest that obesity may spread within social networks in a quantifiable and discernible pattern that depends on the nature of social ties [11]. Therefore, we acknowledge that the influence of the child’s social context (i.e. the extended family, school, friends and peers) is of importance in family-targeted interventions.

Several studies have investigated the barriers to achieving behavior goals and participating in healthy lifestyle programs in obese adolescents [12–15] and parents of obese children [16, 17] as well as the factors that facilitate this. Examples of barriers mentioned include the stigma associated with overweight or obesity, unsafe neighborhood in which to engage in physical activity, lack and inconvenient location of available health services, broader social family barriers as well as time and financial costs. Facilitators mentioned include the subsidization of program costs, enhancement of motivation for adolescents and parents, on-going follow up, treatment offered at convenient locations on a school-friendly schedule and family involvement.

However, these studies did not take the expectations of the child and parents into account. Qualitative studies of young (<12 years) overweight or obese children and their parents that are participating in a behavioral family lifestyle intervention program are scarce. In their systematic review, Lachal et al. highlight the value of qualitative studies in increasing our understanding of the experience of the obese child [15].

In order to improve and optimize future behavioral family lifestyle intervention programs, a greater understanding of the perceptions of obese children and their parents is required. As indicated by previous research, the influence of social factors, family expectations and the capacity of families to change behavior in response to childhood obesity treatment have not yet been properly studied. With these recommendations in mind, the aim of our qualitative study is to acquire more insight into the perceptions of parents and children with regard to their expectations of and experiences in lifestyle interventions and the influence of the social context and social factors on these interventions.

## Methods

We used the consolidated criteria for reporting qualitative research (COREQ) to describe our methods [18]. These criteria describe three domains: 1) research team and reflexivity (including the subdomains ‘personal characteristics’ and ‘relationship with participants’); 2) study design (including ‘theoretical framework’, ‘participant selection’, ‘setting’ and ‘data collection’); and 3) analysis and findings (including ‘data analysis’ and ‘reporting’).

### Design

A qualitative study was conducted on the basis of semi-structured interviews. Interviewees were selected from three different lifestyle intervention programs carried out in different settings (youth health care, primary health centre and paediatrics) in the Northwestern region of the Netherlands. The interventions varied between an extra consultation (youth health care) and a 6- (paediatrics) or 9- (primary health centre) month lifestyle programs, with weekly sessions under the supervision of a physiotherapist or dietician. Participation in the interventions was initiated either by a referral from a school nurse/doctor, general practitioner or pediatrician or by the participant him or herself. All three programs were designed to alter lifestyle behavior by changing eating habits or physical activity and prevent additional weight gain. In all programs, parents were actively involved either during consultations or during separate parent sessions. The aim of the study was to obtain information from a varied group of obese children and their parents to gain insight into their individual experiences. The Medical Ethical Committee of the VU University Medical Center approved the study protocol (METC number 2010105).

### Participants and procedures

In the youth health care setting, overweight and obese children and their parents received an informal, face-to-face invitation to participate in the study by the school nurse, during or after a (regular) consultation. Fifteen parents of overweight or obese children from a primary health centre and eight parents of overweight or obese children from a hospital paediatric department received a letter inviting them to participate within 1 year of the start of the lifestyle program. Sufficient knowledge of the Dutch language was used as a selection criterion when inviting parents and their children to participate. The majority of the participating children in the three interventions were between 4 and 12 years of age. Children were asked to participate by their parents. As an incentive, each participating family received a 10-Euro gift voucher.

Based on the literature, the research team developed an interview guide (Additional file 1: Table S1) and observational protocol (Additional file 1: Table S2) with a main focus on the expectations and needs of overweight and obese children and their parents, the factors that facilitate success in the programs as well as the barriers they face. Examples of questions asked include “What were your expectations of the program?” and “How did you implement the lessons you learned in your daily life and which elements motivated you and which did not?”. After an evaluation of the first two interviews, increased attention was devoted to the possible supporting role of the general practitioner in a lifestyle intervention program.

### Data collection

Data collection took place between January and May 2010. Two researchers (AS, GS) who are trained vocationally in general practice conducted the interviews. One of the researchers who was primarily responsible for the interviews (AS) participated in a course on qualitative research and provided instruction to the other interviewer (GS). All data coders/analysts were trained in qualitative research.

Children and parents were interviewed during the day, at home. The child was always the first to be interviewed. We tried to interview the child and parent(s) separately. In some cases, this was not possible because some children did not want to be interviewed alone and in some instances the parents were not able to sit in another room. When both parents participated in the interview, they were interviewed together. To increase confidence, each interview with a child started with a general question regarding their experiences at school. At the end of the interview, the interviewer checked whether or not all topics had been covered [19]. Each interview with the parent(s) or child lasted between 30 and 60 min. We continued interviewing until no new information emerged. All interviews were audio recorded and transcribed verbatim. Written and verbal consent were obtained from the child and at least one parent, which also covered the recording and transcription of generated data. In addition, demographic information and field notes/observations were made at each interview (Additional file 1: Table S2).

### Data analysis

Transcripts were coded thematically to systematically analyze the data using the Atlas.ti 5.2 software package (www.atlasti.com). First, open coding was conducted (AS). This involved reading and re-reading the transcripts and grouping data into text segments. Two researchers (MvS, LdV) independently checked for coding consistency and reached a consensus (with AS) on a preliminary set of codes. For further analysis, grouped data was compared to explore thematic patterns, using a matrix. To increase inter-observer reliability, transcripts were then re-read and themes and interpretations were critically discussed by five members of the research team (AS, MvS, LdV, LV, SB). The primary themes that emerged from the analysis were: expectations, parenting, social context and needs. The final step in the analysis was the discussion of these findings by AS and MW and the identification of possible explanations. The participants did not provide feedback on the findings.

## Results

### Participants

A total of 18 children and 24 parents (17 mothers, seven fathers) participated. Half of the families invited from the primary health centre and the hospital pediatric department participated. The reasons for declining participation are not known. Parents and children were invited to participate via an informal recruitment procedure at the youth health care level. As such, response rates cannot be reported. In total, seven children from youth health care participated.

### Participant/family characteristics

Table 1 presents information on the characteristics of the participating families by program. The mean age of the children was 10 years (8–15 years) and 56 % were female. The mean age of the parents was 41 years (30–51). In 11 interviews (61 %) only the mother was interviewed; in one interview (6 %) only the father was interviewed and for the rest (33 %), both parents were interviewed. With the exception of the interview with one mother whose older daughter translated during the interview, all parents and children spoke sufficient Dutch. All the children from YHC were participating in the intervention at the time of the interviews. In contrast, the children from the paediatrics intervention had all finished the program. Two children (29 %) from the primary health centre dropped out of the intervention and three had completed it (43 %). There were three one-parent families (17 %). In ten families (56 %), both parents had paid employment. Of all participants, eight children (44 %) had experience with another, previous lifestyle intervention program. After completing the current lifestyle program, nine children (50 %) were still motivated to lose weight.

**Table 1:** Family characteristics by program

*Program*	*N*	*Gender (n female)*	*Age (range years)*	*Ethnicity*	*Highest education*	*Weight problem since (range years)* ^*a*^	*Weight loss after LP (n yes)* ^*b*^	*Motivated to lose weight after LP (n yes)* ^*c*^
Primary health centre	*7*	*4*	*9–15*	3 Dutch	7 Secondary school	*3–5*	*2*	*3*
				2 Moroccan				
				1 Surinamese				
				1 Turkish				
Youth Health care	7	3	7–11	2 Dutch	7 Secondary school	1–4	2	3
				2 Moroccan				
				3 Turkish				
Paediatric	4	3	8–12	4 Dutch	2 Secondary school	2–5	3	2
					1 College			
					1 University			

^a^Weight problem since – range of years that the child has had a weight problem at the time of initiation of the interview

^b^Weight loss after current lifestyle program (LP)

^c^Motivation of child to lose weight after current lifestyle program (LP)

### Themes

The following themes: expectations, barriers and facilitators in terms of parenting and social context and needs are described. Additional file 1: Table S3 provides in-depth information per interviewed family. A brief summary of the aforementioned issues present in each family is included.

### Expectations

All parents and children involved at the level of the primary health centre or hospital programs expected to lose weight in the lifestyle program by being physically active and learning about healthy food.

More than half of the parents and children who were in the youth health care group mentioned expectations. No differences in expectations were found between parents and children involved in a lifestyle program for the first time as compared to the more experienced parents and children.

“My idea was that she would exercise and do sports and receive consultations from the dietician and after a month I would see my daughter slim, but unfortunately that did not happen.” (*Mother ID 2).*

Parents and children expressed hope that bullying would stop due to weight loss.

“I want to have a healthy child. My child stopped playing football on the street. He was being bullied because he could not run due to his weight. I don’t want him being bullied because of his weight.” (*Father ID 11).*

Although parents and children expected their child to lose weight, not everybody managed to do so during or after completion of the lifestyle program. Moreover, some mentioned high drop-out rates during the program.

“At the start, more children were present than at the end. It was unclear why these children did not finish. I think it was only my daughter that lost weight because she was the only one who received a certificate.” (*Father ID 6).*

Parents and children indicated the lessons they learned in establishing and adhering to rules to change the child’s lifestyle behavior. They mentioned their daily routine and eating pattern as being one of the things they had to change, by eating breakfast on a regular basis, for example.

“I don’t like to get up in the morning, I have to get dressed, brush my teeth, wash my face […] You have to go to school again and learn things. I only like to see my friends. So after getting up, I have to comb my hair and watch TV for 10 min. Sometimes I don’t eat breakfast because I wake up too late. Since the program, I’ve been trying to eat breakfast more often. I always have to eat breakfast alone because my mom and sister leave the house at 7.30 am and my dad leaves at 5.30 am.” *(ID 6).*

### Barriers and facilitators

Parenting and the social context (i.e. extended family, school, friends and peers) were the two areas in which barriers and facilitators were experienced.

### 1: Parenting

Parents struggled most with the introduction of new rules. They experienced difficulties with being consistent and dealing with the continuous conflicts with their child while trying to adhere to the rules.

“It is hard when my child doesn’t listen to me and doesn’t want to eat any fruit.” *(Mother ID 2).*

For mothers the conflicts with their child were particularly hard to deal with, as they did not get support from their partner.

“According to my husband, there was no need for this program. He thinks it is all nonsense.” *(Mother ID 13).*

In addition to the issues described above, mothers mentioned their own struggles with temptation to eat unhealthy food such as sweets and chips and the difficulty associated with preventing their child from eating these items.

“During the day he has no appetite and then at night he is hungry and I’ll make sandwiches for him because I feel sorry for him […] In the past, I set the wrong example because I would eat at irregular hours, so he saw me eating and then he wanted to have something too. I gave him everything and the portions kept getting bigger […] We have to do it together, but it was difficult […] during the program my husband was abroad and I had to do it myself.” *(Mother ID 4).*

The new rules were difficult for parents to apply during visits from family members who expected a lot of tasty and largely unhealthy food, which violated the new rules. It was difficult to prohibit the eating of unhealthy food in these situations.

Interviewer: “Who are the key people that should tackle the weight problem?“Respondent: “I hope my wife, with cooking. My parents, they live next to us, they have changed a little bit. My son gets everything he asks for from his grandfather. His grandfather loves him, he is the first grandchild.” (*Father ID 11).*

Parents also indicated that tempting (TV)-advertisements for snacks, candy and soft drinks did not help the children adhere to the rules.

“Then there is an advertisement for candy, then one for ice-cream and children want to try everything. It is hard to deal with the media.” (*Mother ID 16).*

Overweight parents found it easier to be consistent in their parenting when they had insight into the emotional consequences of being an overweight child based on their own experience in their youths.

“I was an overweight child myself. So of course I feel sorry for her. Her length size is 40, but in terms of width I should get 50. It’s annoying for her and Monday she is officially a teenager. (*Mother ID 16).*

According to the children, the new rules introduced by their parents were inconsistent, e.g. rules existed for using the pc, but not for watching TV; they were not allowed to eat candy after four o’clock, but they had ice cream for dessert. Children indicated that it was difficult to stick to the rules when soft drinks and chips were available in the house.

The support of their parents in terms of reminding them not to eat unhealthy food and to go outside and play helped children to overcome these problems.

“It helps me when my mother says go out outside to play and stop watching TV.” *(ID 18).*

“My father and I are alike and I also eat too much. So now we do sports together and I’ve changed my portion size just like my father did because it helped him.*” (ID 8).*

### 2: The social context

In their social context, parents and children struggled with the new rules as well.

It was difficult to prohibit unhealthy food when the child visited family members who gave the child food that was restricted at home.

“I try to change food habits at home, but then I visit my mother and all the unhealthy food is present. It remains difficult. I would have to change the my relatives’ behavior, but that’s impossible.” *(Mother- ID 7)*

When parents lacked support from their own family members, they benefitted from receiving support from other parents in the lifestyle program. Examples of experienced support given include recognition, sharing of successes, exchanging experiences and sharing tips and tricks for raising children.

Parents felt more confident when they received support from their peers. In an effort to create a forum for this type of support, one parent created a social network site at the end of one of the lifestyle programs.

“It is really important to speak to other parents. During the evaluation we also agreed that more parent meetings should be planned. The support you get starts when you get to know each other in the program […] It is important to know you are not the only one and to hear that other parents are also dealing with problems. For me it was enlightening and surprising to find out how long children watched TV or played computer games because that was not an issue for us […] And when things are not going very well, you can discuss this with each other.” *(Mother ID 14).*

In cases in which the parents were informed that their obese child was being bullied at school, they indicated being motivated to support their child losing weight. Schools could be more supportive of the new rules regarding healthy food by choosing healthy treats.

Children struggled at school with being bullied and dealing with slim peers. This made it difficult to change their lifestyle behavior. They also indicated the existence of a stigma associated with being overweight. For example, some felt embarrassed by their body during swimming lessons. Children experienced other problems with friends, mainly slim friends, related to the difficulty of saying no to candy or energy drinks that friends would bring along. On the other hand, friends and peers could help stimulate change. Some received support from their friends, mainly overweight friends, by talking about the problems they experienced. The same support was experienced by the peers in the program and was brought about by a feeling of equality.

### Needs

Parents realized that changing lifestyle behavior requires a continuation of the program.

“It is hard to maintain the changes because after the program, there is no reminder anymore. You miss the feedback that you are on the right track.” (*Mother ID 14).*

One solution mentioned by parents to tackle this problem is the continued involvement of the general practitioner. The general practitioner should play an active role not only in signalling the weight problem in time, but also in offering ongoing support.

“It would have been nice if the GP would have asked how we were doing during the program. That would have been an extra stimulant for him.” *(Mother ID5).*

Another need mentioned was financial support. Parents indicated that financial problems impede lifestyle behavior change. Similarly, they identified the need to be made aware of cheap sports facilities nearby.

Children identified a need for the support of the general practitioner in identifying the weight problem in time, in a non-offensive manner and specifically in providing information on the long-term consequences of obesity.

“I want the GP to be honest with me and tell the truth about my weight. Now I know what can happen and I can do something about it.” *(ID 8).*

## Discussion

In this study, the expectations and needs of overweight and obese children and their parents and the factors that facilitate and prevent success in making and sustaining lifestyle behavior changes were explored. The results show that parents and overweight and obese children themselves expected that they would lose weight by being physically active or by eating more healthily. Barriers to a healthylifestyle were associated with parenting problems, specifically pertaining to the adoption of and adherence to new rules. Both parents and children lacked support from their (extended) family members. Within their social context, children struggled with being bullied at school. For both parents and children, support from (other overweight or obese) peers is encouraging. To maintain lifestyle behavior change, overweight and obese children and their parents need support from their (extended) family, school, friends, peers and their general practitioner. The general practitioner should play a role in identifying weight problems in time, discussing weight in a non-offensive manner and providing information on the long-term consequences of obesity.

Overweight and obese children and their parents participating in this research project indicated the same barriers to behavioral change as other recent studies have [12–17], namely parenting, difficulties with other family members, duration of the intervention and bullying. In addition, factors that facilitated making lifestyle behavior changes included the positive involvement of parents and the utility of continuing the program.

Parents expected their child to lose weight by being more physically active and by learning more about healthy food. The primary goal of the three programs was to change the participants’ lifestyle behavior by changing their diet and instilling in them an appreciation for being physically active and to prevent further weight gain. Some children and parents were disappointed that they did not lose weight and withdrew from the program as a consequence. Previous research involved the conducting of a survey of respondents from a tertiary care weight management centre and found that parents’ mismatched expectations were important reasons for attrition [20]. It can be speculated that when participant expectations and program goals are better coordinated, the motivation of the participants will increase, leading to a reduction in drop-out rates and eventually, greater long-term success.

In our study, for both children and parents, the main barrier was to set and maintain rules; inconsistent parenting was a problem especially for children. It is known that parenting styles are an important factor determining a child’s health [21, 22]. Parents play an important role in stimulating healthy eating behavior in their children [23]. In previous research on obese adolescents, the intention to reduce weight was interfered with by quarrels with parents, self-blame and misguided understanding of eating and exercising habits [24]. Similarly, previous research reported that recognizing family behavior is essential in the development of weight control and weight loss activities [25]. As in our study, almost all children and parents that were motivated to change eating patterns, succeeded in achieving weight loss. In contrast, it was hard for the children with unmotivated parents to establish a change. Furthermore, the child’s commitment to weight loss and the support of his or her family has been identified as being important to successful weight loss [26]. Another barrier was dealing with advertisements for unhealthy food. It is known that food advertisements contribute to an obesity-promoting environment [27]. Fortunately, many countries are now establishing new regulations on advertising directed toward children on TV and many government health agencies are now issuing recommendations for parents regarding the amount of time children spend watching TV [28].

In their social context, the extended family impeded progress from the standpoint of the parents. In line with previous research, the involvement of the wider family and social network of participants is important to the success of the intervention [10, 11]. Peers were sources of support for both parents and children. As has been shown in previous research, peers can increase a participant’s motivation to be physically active [29]. A barrier experienced by the children was bullying at school. Previous research has concluded that bullying is significantly associated with low self-esteem in overweight children [30, 31].

In order to maintain lifestyle behavior change on the long term, parents and children mentioned the need for the support of the general practitioner. Previous research has shown that primary care is an appropriate setting in which to treat childhood obesity [32]. Parents of obese children were reluctant to consult a general practitioner due to a fear of being blamed for their child’s weight problem and concern about their child’s mental well-being [32].

In a recent systematic review and meta-analysis a small effect size of behavioral family lifestyle interventions was shown [8]. In 19 of the 20 studies included, the same age group was used and, on the basis of 18 studies, we were able to gather information on the background of the given interventions. In 15 interventions, the family (parent and child) was involved in the intervention; three interventions focused only on the parents. In only two studies was special attention given to the expectations of the participant(s). Furthermore, only two studies dealt with social context by sharing tips with other parents and discussing barriers in the environment. In only one study did the general practitioner participate in the intervention.

The current study has some limitations. Our aim was to interview children and parents separately, but in fifteen interviews the parents and children were interviewed together. This may have influenced what children were willing to say in front of their parents. However, we experienced that parents were in fact supportive in terms of helping the children with providing examples of experiences in the lifestyle program. Another limitation is the unknown recruitment rate from the youth health center. However, the recruiting school nurses were asked to pay special attention to recruiting respondents with variability in, age, gender, ethnicity, differences in lifestyle setting and weight loss success. Half of the families invited from the other two lifestyle programs participated. The participating families have characteristics that are representative of the greater population in which obesity is common. However, generalizability is limited to the one region included in the study.

A strength of the study is the participation of children and parents from different types of interventions and xsettings. We paid special attention to the differences between the three interventions and while interpreting the results we only found substantial differences in the expectations between parents and children of the YHC intervention and the other two interventions. This was to be anticipated since not all parents and children knew in advance that an intervention would take place. The setting and type of intervention seemed not to be particularly important, since the same perspectives were identified by both parents and children.

Our results underline the importance of paying attention to the expectations of the participants and discussing barriers and facilitators within the entire social context during an intervention. It is recommended that clinical practice actively inform general practitioners of participants’ involvement in a lifestyle program in order to maintain behavioral change after the program is completed. Future research would benefit from qualitatively evaluating children’s and parents’ experiences with lifestyle programs to improve their adoption and implementation [33].

We can conclude that parents and children need parenting support and help from their (extended) family and social context when attempting to make lifestyle behavior changes. The general practitioner should play a more supportive role.

## Additional files

Additional file 1: Table S1. Interview guide. **Table S2.** Observation protocol. **Table S3.** Themes and summary per family. (DOCX 25 kb)

## Abbreviations

COREQ: Consolidated criteria for reporting qualitative research
