# Supplementary material for: Perspectives of obese children and their parents on lifestyle behavior change: a qualitative study
Source: Int J Behav Nutr Phys Act. 2015 Aug 19;12:102. doi: 10.1186/s12966-015-0263-8 (PMC4539727; doi:10.1186/s12966-015-0263-8)
Supplement: Additional file 1: Table S1. — Interview guide. Table S2. Observation protocol. Table S3. Themes and summary per family. (DOCX 25 kb) [file 12966_2015_263_MOESM1_ESM.docx]

**Table S1: Interview guide**

Each interview started with a general introduction and an explanation of how we collected their personal information. The name and setting of the lifestyle intervention program of the child was mentioned. After this brief introduction, the interviewer would introduce herself and thereafter the child was asked to introduce him-/herself.

Subsequently, the interviewer continued the conversation with the child/parent by discussing the following topics using the questions below. The order of the questions could vary, depending on the way the interview proceeded.

| ***Topics*** | ***Questions*** |
| --- | --- |
| Participating Program | - - How did you learn about the existence of the program? Were you referred?   - What were your expectations of the program?   - What was your experience of the program?   - What was the duration of your participation in the program?   - How/to what extent did you maintain the things you learned in your daily life?     - Which elements were motivating/ which were demotivating?   - How did you perform the program?     - Alone or with the rest of the household?     - Who do you think was responsible for the outcome after participation (child, parent, other?) |
| Expectations | - - What were your weight loss expectations?   - What was your role during the program?   - What was your expectation of the treatment given by the caregiver/general practitioner?   - What would you expect from a new lifestyle intervention? |
| Consequences overweight / obesity | - - What was your perception of your own weight (child)?   - What were the causes of your overweight/obesity?   - What did you know about healthy food, drinks and exercise?   - What was the role of the general practitioner in your overweight/ obesity problem? |
| Future | - - What should the role of the general practitioner be in managing childhood obesity?   Parent:   - - Would you consult a parenting expert, why (not)?   - Would you be prepared to exercise with your child, why (not)? |

**Table S2: Observation protocol**

| - What was the duration of the interview? - Other people besides child and parent(s) present at the interview? - Respondent(s) fluent in Dutch? - Which questions were difficult to answer and why? - What are the social skills of respondent(s)? - In what kind of area did the respondent(s) live:   - Type of house?   - Same houses in the neighbourhood? - How did the interview proceed? - Which topics were interesting to the respondent(s)? - What was the general level of cooperation of the respondent(s)? - Were there other obese people in the household? - Was the child still looking overweight/obese? - Where was the television located? - Was the television turned on upon arriving/ during interview? |
| --- |

**Table S3: Themes and summary per family**

| **Theme** | | **Expectations** | | **Parenting** | | | | **Social context** | | | | **Needs** | |
| --- | --- | --- | --- | --- | --- | --- | --- | --- | --- | --- | --- | --- | --- |
|  | |  | | **Facilitators** | | **Barriers** | | **Facilitators** | | **Barriers** | |  | |
| **Id** | |  | |  | |  | |  | |  | |  | |
| 1 | | Lose weight by eating less and being more physically active. Mother did not expect to have a role herself during the program. During the program learned what to eat and was physically active. After program girl eats less, but did not lose weight. | | Introduced rules for healthy eating in the family.  Snacks and chips are less present in the house. | | Experienced difficulties adhering to these rules, especially due to the pressure felt from other visiting family members.  Family members’ expectations for plenty of food during family visits. | | Discuss girl’s weight problems with friends. | | Child only obese girl in the family. | | Support from the GP.  Consulted the GP frequently, but did not find support. The GP rejected request for referral. | |
| 2 | | Discussion of (non)healthy food and what to change to eat healthy. Be physically active and (hopefully) lose weight. | |  | | Could not manage to be strict in applying the rules due to long working hours.  Felt guilty for working hard resulting in the girl often being home alone.  Girl did not feel supported by her mother. | |  | | Girl did not feel supported by friends/peers in changing her lifestyle.  Peers in lifestyle were younger. | | Lifestyle program may be followed after school time and not within school hours. | |
| 3 | | Expected the program to be an incentive for the boy to change his lifestyle. | |  | | Handling behavior of son who wants to eat all day long. | |  | | Program did not help and the boy was rejected at the end of the program.  Support from general practitioner and other health care providers, therefore mother is despondent. | | Support from GP. | |
| 4 | Lose weight by being physically active and eating less. | | Mother has experience with being obese. | | Difficulty in adhering to rules due to missing consensus in parenting and quarrels with boy.  Boy not motivated and did not obey rules at home or at program. | | Support from GP. | | Lacked support from extended family. Extended family still buys sweets and snacks. | |  | |  |
| 5 | Lose weight by being physically active and talking about food and activities. Boy still conscious of food. | | Proud of boy he managed to lose weight.  Mother respected boy since she had own difficulties with losing weight.  Changed rules like schedule dinner later to avoid snacking.  Boy motivated to lose weight since he learned about the consequences of being overweight over the long term. | | Tempting TV-adverts difficult to deal with. | | Support from parents and peers.  Certificate after program. | |  | | Support from the GP.  Continuity of program as a reminder. | |  |
| 6 | Lose weight by being physically active and learn about healthy food. Did not expect that she would make friends at the program. Girl likes sweets and realized she likes sugar. After program eats fewer sweets and eats breakfast. | | Proud of girl she managed to lose weight and maintain physical activity.  Motivated to change rules in the family, like eating breakfast and trying to abandon sweets.  All member of the family took part at the program.  Girl motivated to be physically active since she learned cause of being overweight.  Support from parents. | |  | | Support from peers. | |  | |  | |  |
| 7 | To be slim, like classmates so that bullying stops and to buy new clothes.  Mother felt helpless when starting the new program due to previous negative experiences with lifestyle programs and problems at school. | |  | | Girl lacked self-confidence. Attending program had to be secret kept from friends, due to school problems. | |  | | Tried to involve family, however cultural practices interfered. Lacked support from the extended family and the school. | |  | |  |
| 8 |  | | Mother motivated to change lifestyle, she knew consequences of childhood overweight over long term.  Received support from parents.  Boy motivated to lose weight, he knew the cause of being obese, namely eating too much. | |  | | Mother changed cooking pattern and involved extended family.  Social support was important.  Support from the GP. | |  | |  | |  |
| 9 | Mother did not change behavior, since it is too hard. | | Mother changed rules about soft drinks and sweets as a treat, since she was shocked that both her children were obese. | | Girl not motivated and did not feel support from mother. | |  | |  | |  | |  |
| 10 |  | | It helped boy to realize that it is for his own good to sport and eat healthy.  Support from mother. | | Mother felt guilty for boy being obese since she was not able to be strict.  She could not manage to maintain the rules.  Support from extended family was lacking.  Boy did not like to be obese and knew the reason he became obese, namely eating too much. | | Support from friends. | | Mother worried about son not getting married in the future due to obesity. | |  | |  |
| 11 |  | | It helped father to think about the future of his child. He wants a healthy child without diseases like diabetes.  Father was very motivated to introduce rules and change shopping behavior in not buying chips and soft drinks.  As a family they were aware of the causes of and problems associated with obesity.  Support from parents. | | Grandparents play a role in pampering the boy.  Rules were needed, but hard to introduce in the extended family. | |  | |  | |  | |  |
| 12 | Lose weight by being physically active and eating less. | |  | | Help and support from parents to find a sport.  Soft drinks and sweets available at home. | |  | |  | | Information on (cheap) sports facilities nearby.  Support of GP. | |  |
| 13 | Lose weight by being physically active and eating less. | | Support of mother.  More aware of shopping behavior and involving girl.  Tips and tricks for indoor physical activity. | | Lacks support of partner. | | Support by paediatrician at program and compliments for performance.  Support by peers. | |  | | Continuity of program for a helping hand. | |  |
| 14 |  | | Change shopping behavior and introduction of rules, introduced a week scheme.  Support of mother. | |  | | Support by friends, when girl makes own decision what to eat.  Support peers. | | Slim friends. | | Signalling the weight problem in time by the GP. | |  |
| 15 | Lose weight by being physically active and eating a special diet. | | Support of mother. Reminding him to go outside to play. | | Boy does not obey mother’s rules.  No support from partner. | | Support by YHC by giving information of consequences on overweight. | | Bullying at school. | | Teach children to respect parents and obey. | |  |
| 16 | Lose weight by being physically active and eating differently. | | Support within the family.  Mother supported girl, since she had own difficulties with losing weight.  Introduction of rules for pc-use. | | Temping TV-adverts difficult to deal with. | |  | | Bullying at school. | | Financial support for sports.  Support from the GP.  Signalling the weight problem in time by the GP.  Continuity of program for a helping hand. | |  |
| 17 | Learn about consequences of being overweight. | | Support within the family. | |  | | Support from peers. Support from extended family. | | Bullying at school. | | Continuity of program for a helping hand.  Awareness for children. | |  |
| 18 | Being physically active and eating less, grow and weight stabilizes. | | Support within the family.  Results during and after the program. | |  | | Support from peers. | | Bullying at school. | | Signalling the weight problem in time by the GP.  Support from the GP. | |  |
